# Targeting working memory to modify emotional reactivity in adult attention deficit hyperactivity disorder: a functional magnetic resonance imaging study

**DOI:** 10.1007/s11682-021-00532-6

**Published:** 2021-09-15

**Authors:** Antonia Kaiser, Liesbeth Reneman, Paul J. Lucassen, Taco J. de Vries, Anouk Schrantee, Anne Marije Kaag

**Affiliations:** 1 Department of Radiology and Nuclear Medicine, Amsterdam UMC, University of Amsterdam, Amsterdam Neuroscience, Location AMC, Meibergdreef 9, 1105AZ Amsterdam, The Netherlands; 2 Brain Plasticity Group, Swammerdam Institute for Life Sciences, Center for Neuroscience, University of Amsterdam, Amsterdam Neuroscience, Amsterdam, The Netherlands; 3 Department of Anatomy and Neurosciences, Amsterdam UMC, Vrije Universiteit Amsterdam, Amsterdam Neuroscience, Amsterdam, The Netherlands; 4 Department of Psychiatry, Amsterdam UMC, University of Amsterdam, Amsterdam Neuroscience, Amsterdam, The Netherlands; 5 Department of Developmental Psychology, University of Amsterdam, Amsterdam Neuroscience, Amsterdam, The Netherlands; 6 Neuro- and Developmental Psychology, Department of Clinical Neuropsychology, Vrije Universiteit Amsterdam, Amsterdam Neuroscience, Amsterdam, The Netherlands

**Keywords:** Adult Attention-Deficit/Hyperactivity Disorder (ADHD), Emotional dysregulation, Emotional reactivity, Functional magnetic resonance imaging (fMRI), Working memory

## Abstract

Understanding the neural mechanisms of emotional reactivity in Attention-Deficit/Hyperactivity Disorder (ADHD) may help develop more effective treatments that target emotion dysregulation. In adult ADHD, emotion regulation problems cover a range of dimensions, including emotional reactivity (ER). One important process that could underlie an impaired ER in ADHD might be impaired working memory (WM) processing. We recently demonstrated that taxing WM prior to the exposure of emotionally salient stimuli reduced physiological and subjective reactivity to such cues in heavy drinkers, suggesting lasting effects of WM activation on ER. Here, we investigated neural mechanisms that could underlie the interaction between WM and ER in adult ADHD participants. We included 30 male ADHD participants and 30 matched controls. Participants performed a novel functional magnetic resonance imaging paradigm in which active WM-blocks were alternated with passive blocks of negative and neutral images. We demonstrated group-independent significant main effects of negative emotional images on amygdala activation, and WM-load on paracingulate gyrus and dorsolateral prefrontal cortex activation. Contrary to earlier reports in adolescent ADHD, no impairments were found in neural correlates of WM or ER. Moreover, taxing WM did not alter the neural correlates of ER in either ADHD or control participants. While we did find effects on the amygdala, paCG, and dlPFC activation, we did not find interactions between WM and ER, possibly due to the relatively unimpaired ADHD population and a well-matched control group. Whether targeting WM might be effective in participants with ADHD with severe ER impairments remains to be investigated.

**Supplementary Information:**

The online version contains supplementary material available at 10.1007/s11682-021-00532-6.

## Introduction

In addition to deficits in attention, hyperactivity, and impulsivity (American Psychiatric Association, 2000), emotional dysregulation (ED) is considered a core symptom in adults with Attention-Deficit/Hyperactivity Disorder (ADHD) (Hirsch et al., 2019). ED is defined as the inability to control and minimize the disrupting effects of irrelevant emotional stimuli on goal-oriented processes (Barkley & Fischer, 2010; Wehmeier et al., 2010). In adult ADHD, problems with emotion regulation include emotional recognition, emotional responsivity, and emotional lability, adding to the complexity of the spectrum of classic symptoms (Beheshti et al., 2020). Importantly, ED predicts lower quality of life in young adults (Groenewold et al., 2013) and is associated with the persistence of ADHD into adulthood (Barkley & Fischer, 2010). A recent meta-analysis demonstrated that pharmacological treatments have limited efficacy for ED in adults (Lenzi et al., 2018), yielding therapeutic challenges. As such, better insights into underlying (neural) mechanisms of ED in adult ADHD could help develop more effective treatments.

A critical aspect of ED in ADHD is impaired emotional reactivity (ER): the threshold, intensity, or duration of affective arousal, which can be measured through the processing of emotionally salient stimuli (Graziano & Garcia, 2015). Adult participants with ADHD do not appear to have deficits in the explicit regulation of emotions, but display emotional hyper-responsivity (Materna et al., 2019). For example, higher emotional lability has been associated with a hyper-connectivity of the cortico-amygdalar network, including the anterior cingulate cortex, both in children and adolescents (Hulvershorn et al., 2014; Hafeman et al., 2017). In children with ADHD, the processing of negative emotional faces stimuli has been associated with amygdala hyper-connectivity and hyper-reactivity (Brotman et al., 2010; Posner et al., 2011a, 2011b; Quinlan et al., 2017); and was notably also linked to ED (Herrmann et al., 2010). In adults with ADHD, amygdala hyperactivity has further been demonstrated in response to salient stimuli (Maier et al., 2014; Plichta et al., 2009; Tajima-Pozo et al., 2018), but divergent findings have been reported as well (Hägele et al., 2016; Tajima-Pozo et al., 2015) and the exact neural mechanisms that underlie ER in adult ADHD thus remain still unclear.

Emotional regulation has been shown to be influenced by top-down cognitive control processes. For example, in controls, the prefrontal cortex (PFC) was activated stronger in the presence of emotional stimuli during cognitive control tasks (Hung et al., 2018; Song et al., 2017) and cognitively demanding tasks could tune down amygdala reactivity to emotional stimuli, suggesting top-down suppression of ER (Erk et al., 2007; Van Dillen et al., 2009). Furthermore, taxing WM during or prior to the exposure of emotionally salient stimuli reduced ER in both anxiety and substance use disorders (Andrade et al., 2012; Kaag et al., 2018; Markus et al., 2016; May et al., 2010; McClelland et al., 2006; van den Hout et al., 2014). Additionally, WM training has been shown to improve ER outcomes in healthy individuals as well as in individuals with psychiatric disorders other than ADHD (Barkus, 2020; Schweizer et al., 2013). Whether this affects (the neural mechanisms underlying) emotional processing remains to be determined. Indeed, an underdeveloped working memory (WM) system may underlie impaired ER in ADHD (Groves et al., 2020). More specifically, ADHD participants perform worse (Marx et al., 2011) and show reduced WM-related PFC activation in WM-tasks (Cortese et al., 2012; Burgess et al., 2010; Ko et al., 2013). Altogether, this indicates that while WM and ER are strongly related, the underlying neural mechanisms of how emotional and WM processes interact in ADHD are still unclear (Tsai et al., 2020,2020).

Therefore, this study aims to test whether targeting WM processes can reduce ER-related neural activity in adult ADHD, through top-down PFC suppression of amygdala hyperactivity. In order to disentangle neural mechanisms of emotional and WM processes, we used a novel functional magnetic resonance imaging (fMRI) paradigm, interleaving emotional stimuli with WM-load blocks. We expected that ADHD participants, compared to controls, would show higher levels of amygdala activation in response to negative emotional, relative to neutral images. Moreover, participants with ADHD were expected to show decreased dorsolateral PFC (dlPFC) and paracingulate gyrus (paCG) responses to high versus low WM-load tasks. We furthermore expected amygdala reactivity in participants with ADHD to be reduced in response to negative emotional blocks following high WM-load more so than in controls.

## Methods and material﻿s

### Participants

Thirty adults with ADHD and 30 controls (19–35 years of age) were included in the study. Inclusion criterion for the ADHD group was prior clinical ADHD diagnosis according to the DSM-IV (American Psychiatric Association, 2000); controls were excluded with a score > 4 on the ADHD Rating Scale (ADHD-RS) (Kooij et al., 2008). Controls were matched to the participants with ADHD, based on age, educational-level, tobacco use (Fagerström Test for Nicotine Dependence)(Heatherton et al., 1991), alcohol use [Alcohol Use Disorders Identification Test (AUDIT)] (Saunders et al., 1993), cannabis use [Cannabis Use Disorders Identification Test (CUDIT)] (Adamson & Sellman, 2003), and the use of additional substances [Drug Use Disorders Identification Test (DUDIT)] (Berman et al., 2005). Medicated participants with ADHD (N = 12) were instructed to refrain from ADHD medication use for seven days before the MRI scan. Exclusion criteria were: history of brain trauma, neurological disease, excessive consumption of alcohol (AUDIT > 12), cannabis (CUDIT > 12) or other drugs (DUDIT > 12), and MRI contra-indications. For control participants: psychiatric disorders for which they had ever received treatment; for participants with ADHD: psychiatric disorders other than ADHD for which they received treatment at the moment of the experiment. Anxiety, depression, and impulsivity were assessed using the State and Trait Anxiety Inventory (STAI) (Marteau & Bekker, 1992), Beck’s Depression Inventory (BDI) (Beck et al., 1961), and Barratt’s Impulsiveness scale (BIS) (Patton et al., 1995), respectively. Written informed consent was obtained from all participants. The study was approved by the Ethics Review Board of the University of Amsterdam.

### Statistical analyses

Data points more than three standard deviations from the mean were removed as outliers (results before and after outlier removal did not differ). Analyses of task and fMRI data used linear mixed-effects models (lme4 Rv.3.5.3) (Bates et al., 2014; R Development Core Team, 2011). In the case of non-normal distributions, transformations were applied. The main and interaction effects of emotional load, WM-load, and group were assessed as fixed effects. We used an adjusted top-down model selection process using the Bayesian information criterion (BIC) for model comparison (Fabozzi et al., 2014; Schwarz, 1978). The model best capturing the data was reported and compared using χ^2^ tests and BICs. P < 0.05 was considered statistically significant (Supplementary Materials 1.5).

### MRI acquisition

Participants were scanned on a 3 T whole-body MR system (Philips, Best, The Netherlands) using a 32-channel receive-only head-coil. T1-weighted (T1w) scans were obtained using a 3D-TFE sequence (resolution = 0.8mm^3^, FOV = 240 × 256 × 200mm, TR/TE = 9.8 ms/4.5 ms). Functional scans were acquired using a 2D-GE-EPI sequence (resolution = 2.5 × 2.5 × 2.2 mm, FOV = 240 × 240 × 131.8 mm, TR/TE = 1500 ms/30 ms, FA = 70°, MB-factor = 3, SENSE = 1.5). A scan with opposite phase-encoding-direction was used for distortion correction.

### fMRI paradigm

**Fig. 1:**
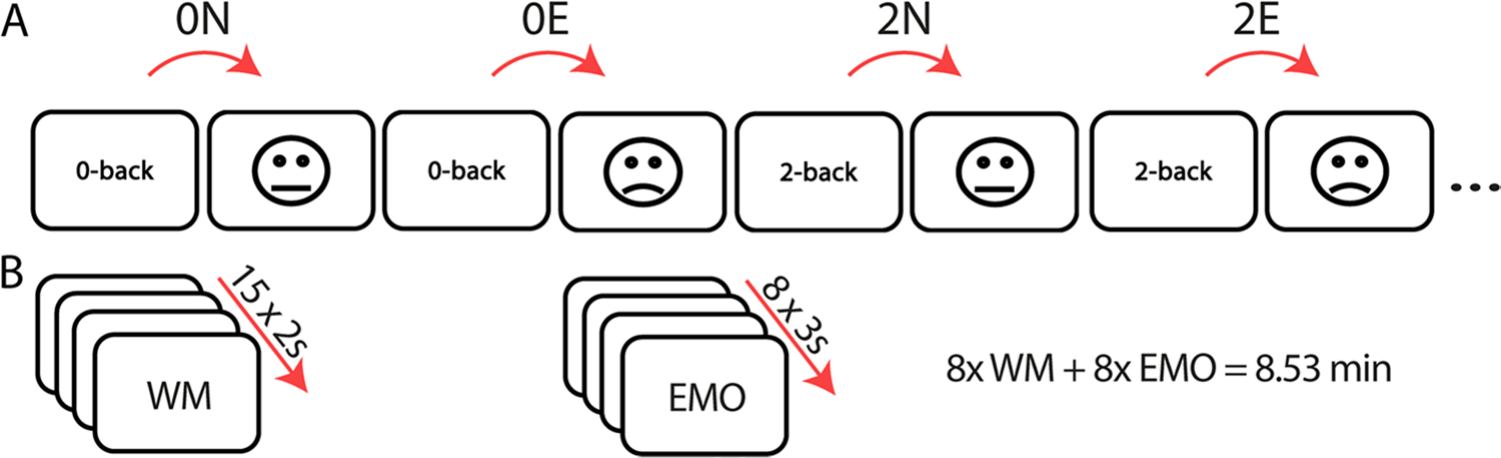
Study design: **A**) Interleaved active n-back blocks and passive emotional stimuli blocks. The effect of WM-blocks preceding emotional stimuli was assessed by subdividing the emotional stimuli into four conditions: neutral-after-0-back (0N), emotional-after-0-back (0E), neutral-after-2-back (2N), emotional-after-2-back (2E). **B**) The working memory (WM) blocks consisted of 15 trials lasting 2s each. The emotional images blocks (EMO) consisted of 8 trials lasting 3s each. Every condition was shown twice, resulting in 8 WM and 8 EMO-blocks randomly interleaved, which results in a total task duration of 8:53 min

The experimental fMRI paradigm (Fig. 1) comprised a blocked design wherein active blocks with either the zero-back or the two-back task (‘WM-block’) were interleaved with passive blocks consisting of either emotionally neutral or emotionally negative pictures (‘EMO-block’). This resulted in four conditions: two-back followed by negative pictures (2E), two-back followed by neutral pictures (2N), zero-back followed by negative pictures (0E), and zero-back followed by neutral pictures (0N). The order of the blocks was randomized per participant, under the conditions that 1) the experiment started with a WM-block and 2) blocks were never immediately followed by the same type.

The WM-blocks consisted of a standard 0-back and 2-back task (Cousijn et al., 2014; Supplementary Methods 1.2). During the emotional block, 64 pictures from the International Affective Picture System (IAPS; Supplementary Materials 1.2) were shown (32 emotionally negative and 32 emotionally neutral; Fig. 1) (Lang et al., 2005). The percentage of correct responses in the n-back task were compared between conditions and groups. All participants performed a recognition task after the fMRI experiment to determine whether both groups paid equal attention to the images during the fMRI-task. Subsequently, participants performed a validation task, in which they rated the valence of all images using the Self-Assessment Manikin (SAM) rating from one (‘negative’) to nine (‘positive’) (Lang, 1980) (Supplementary Materials 1.2–1.3).

### fMRI processing

Preprocessing was performed using FMRIPREP v1.2.3 (Esteban et al., 2019, 2020) [RRID: SCR_016216]. Each T1w scan was normalized to MNI space. Functional data preprocessing included motion correction (FLIRT), distortion correction (3dQwarp), followed by co-registration to the T1w. Independent component analysis (ICA) based Automatic Removal Of Motion Artifacts (AROMA) was used to generate data that was non-aggressively denoised. Subsequently, data were spatially smoothed (6mm FWHM) and high pass-filtered (342s) using FSL (Supplementary Methods 1.5).

FMRI data were entered into the first-level analysis (FSL/FEAT v.6.00; RRID: SCR_002823) (Jenkinson et al., 2012). The model was designed to estimate the effect of WM-load on the neural correlates of emotional processing (Supplementary Materials 1.5). To explore whole-brain activity in the main task contrasts (two-back vs. zero-back; emotional vs. neutral), the first-level contrast-of-parameter-estimates (COPE) maps were analyzed using non-parametric permutation testing (5000 permutations), using FSL Randomise. Thresholds for all analyses were initially set at p < 0.05 with family-wise error corrections using threshold-free cluster enhancement (Winkler et al., 2014). Mean frame-wise displacement per participant was added as a confound regressor.

ROI) analyses used predefined ROIs: Amygdala, dlPFC, and paCG (Supplementary Methods 1.4). The amygdala is a key region in emotional processing (Sergerie et al., 2008), the dlPFC and paCG play an important role in executive functioning, especially during WM-back tasks (Miró-Padilla et al., 2018), whereas the paCG shows overlapping activation during negative affect and cognitive control paradigms (Lin et al., 2015; Shackman et al., 2011). Hemisphere differences were tested using paired t-tests and found to be significant for the dlPFC, but not the amygdala. The left and right dlPFC were therefore analyzed separately, whereas parameters were averaged across hemispheres for the amygdala. Featquery (FSL) was used to extract the COPEs, which were converted to percentage change.

## Results

### Participants

Participants with ADHD and controls were well-matched (Table 1). Two participants were removed from the analysis, due to extreme motion during the fMRI-task (framewise displacement > 1.5 * 95% CI).

**Table 1:** Participant characteristics

		ADHD		controls				
		M (SD)	N	M (SD)	N	Statistics*		
Age (y)		25.18 (4.06)	28	24.40 (3.86)	30	t (56) = − 0.75, p = 0.46		
Education		4.07 (1.63)	28	4.13 (1.63)	30	U = 416.00, z = − 0.06, p = 0.95		
Medication use			12/28					
ADHD-RS								
	*Inattention (child)*	6.71 (2.12)	28	1.33 (1.56)	30	U = 815.50, z = 6.22, p < 0.001		
	*Hyperactivity (child)*	4.57 (2.74)	28	0.47 (0.78)	30	U = 783.00, z = 5.83, p < 0.001		
	*Inattention (adult)*	5.36 (2.78)	28	1.23 (1.19)	30	U = 764.50, z = 5.42, p < 0.001		
	*Hyperactivity (adult)*	3.71 (1.90)	28	1.07 (0.91)	30	U = 752.00, z = 5.26, p < 0.001		
Comorbid psychiatric disorders								
	*MDD*		5		NA			
STAI (trait)		42.19 (10.81)	27	34.10 (5.27)	30	U = 601.50, z = 3.15, p = 0.002		
STAI (state)		38.25 (9.58)	28	30.60 (5.51)	30	U = 621.00, z = 3.13, p = 0.002		
BDI		6.71 (6.26)	28	3.63 (2.99)	30	U = 546.00, z = 1.97, p = 0.049		
BIS		70.00 (9.49)	28	56.43 (6.95)	30	t (56) = − 6.24, p < 0.001		
AUDIT		5.82 (3.74)	28	5.97 (3.36)	30	U = 395.00, z = -0.39, p = 0.67		
DUDIT		2.11 (2.63)	28	1.83 (2.38)	30	U = 441.50, z = 0.30, p = 0.72		
CUDIT		2.50 (4.38)	28	2.40 (3.50)	30	U = 378.00, z = -0.72, p = 0.47		
Tobacco use			11/28		10/30	χ^2^ (1, n = 58) = 0.006, p = 0.94		
Motion (FD, mm)		0.15 (0.07)	28	0.12 (0.03)	30	U = 348.00, z = -1.12, p = 0.26		

*Normally distributed data were tested using independent samples t-tests, otherwise, Mann–Whitney tests, or χ^2^-test were used

Education (Dutch system): 1 = VMBO/VMBO-T; 2 = MBO; 3 = HAVO; 4 = HBO; 5 = VWO; 6 = WO; *ADHD-RS* ADHD-Rating Scale; *STAI* State-Trait Anxiety Inventory; *BDI* Beck Depression Inventory; *BIS* Barrat's Impulsivity Scale; *AUDIT* Alcohol Use Disorders Identification Test; *DUDIT* Drug Use Disorders Identification Test; *CUDIT* Cannabis Use Disorders Identification Test

### Behavioral measures

**Fig. 2:**
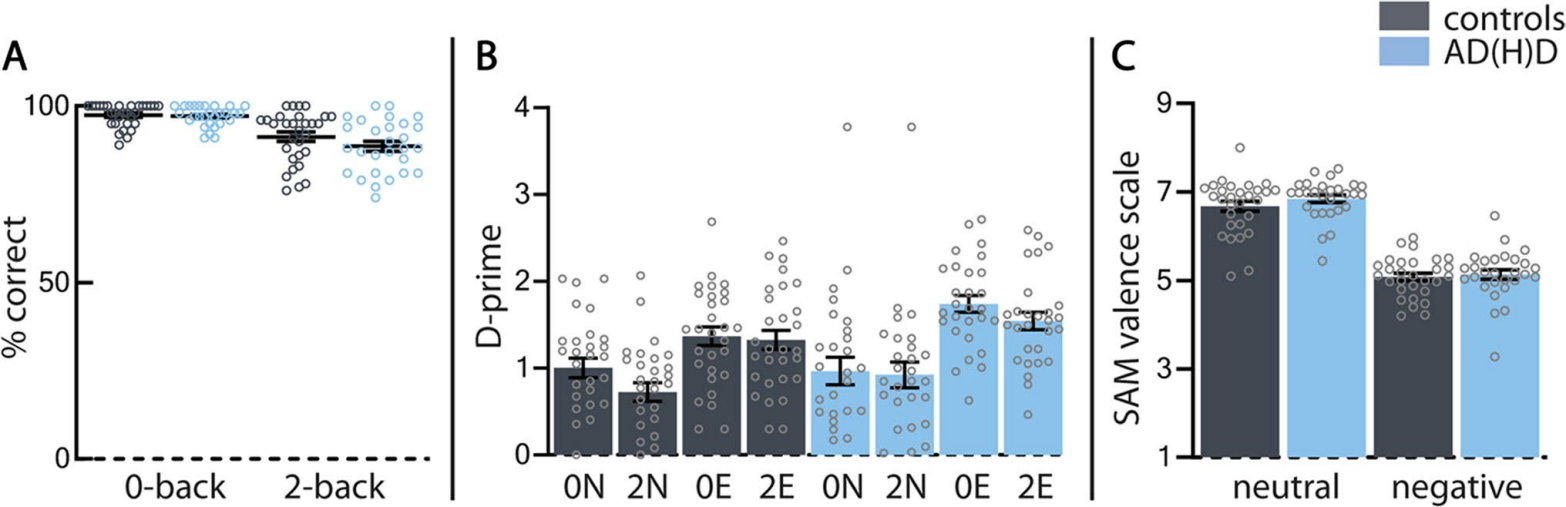
Behavioral data: **A**) Task performance; No significant differences between participants with ADHD (blue) and controls (grey). The performance of the 2-back task was lower than the 0-back task. **B**) Recognition task; Neutral images were recognized less correctly than negative images. There is a trend towards participants with ADHD recognizing negative images better than controls. **C**) Validation task; Both groups rated negative images as more negative than neutral images. Error bars represent the standard error

We found no interaction between WM-load and group on n-back accuracy (χ^2^(1) = 1.68, p = 0.19, ∆BIC =  − 3.07), but a main effect of WM-load was found (χ^2^(1) = 40.29, p < 0.001, ∆BIC = 34.87) (Fig. 2A). The d-prime of the recognition task suggests that more attention was paid towards negative emotional images (χ^2^(1) = 6.78, p < 0.01, ∆BIC = 1.36), and that attention reduced after high-load WM-blocks (χ^2^(1) = 34.87, p < 0.001, ∆BIC = 1.36) (Fig. 2B). Additionally, we found a trend towards an interaction effect of group and emotional image type (χ^2^(1) = 3.68, p = 0.05, ∆BIC =  − 1.74), suggesting that participants with ADHD paid more attention to the negative emotional images compared to controls. The validation task showed that negative images were perceived as more negative than the neutral images by both groups (χ^2^(1) = 143.29, p < 0.001, ∆BIC = 138.55) (Fig. 2C).

### fMRI

**Fig. 3:**
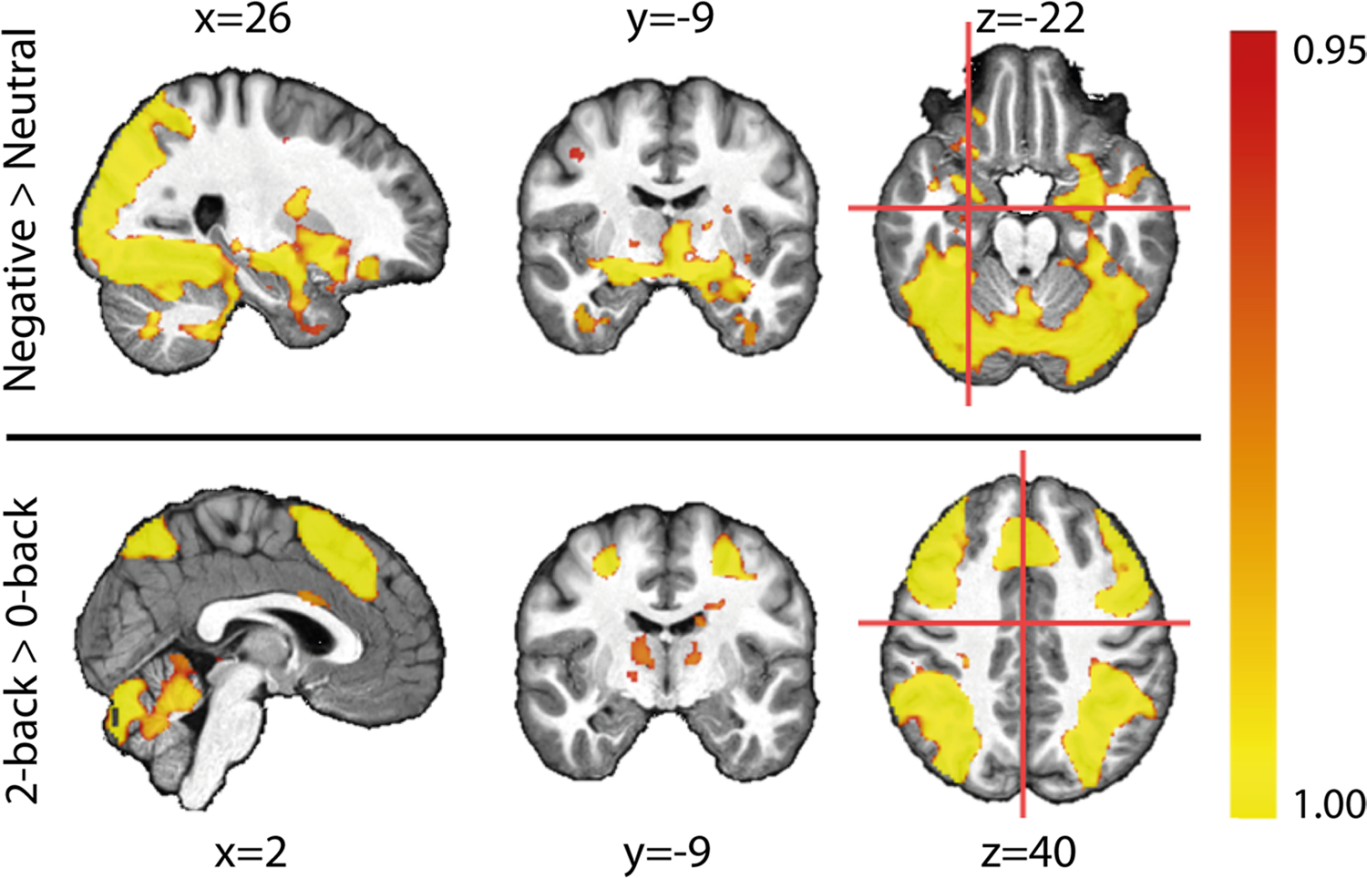
Whole brain activation maps calculated with permutation tests: BOLD signal for negative vs. neutral images (top row) and the 2-back vs. 0-back task (bottom row). Values shown are corrected and therefore correspond to the statistically significant regions. Colors correspond to 1-p results; with red = 0.95 (corresponding to p = 0.05) and yellow = 1.00

Exploratory whole-brain analysis assessed the effects of WM-load, emotional image type, and group. Permutation tests revealed the expected main effects of the WM-load and a main effect of the emotional image type in the executive and salience network, respectively (Fig. 3, Supplementary Results 2.2). In contrast to our hypothesis, we found no main effect of group nor any two- or three-way interactions of WM-load, emotional image type, and group.

**Fig. 4:**
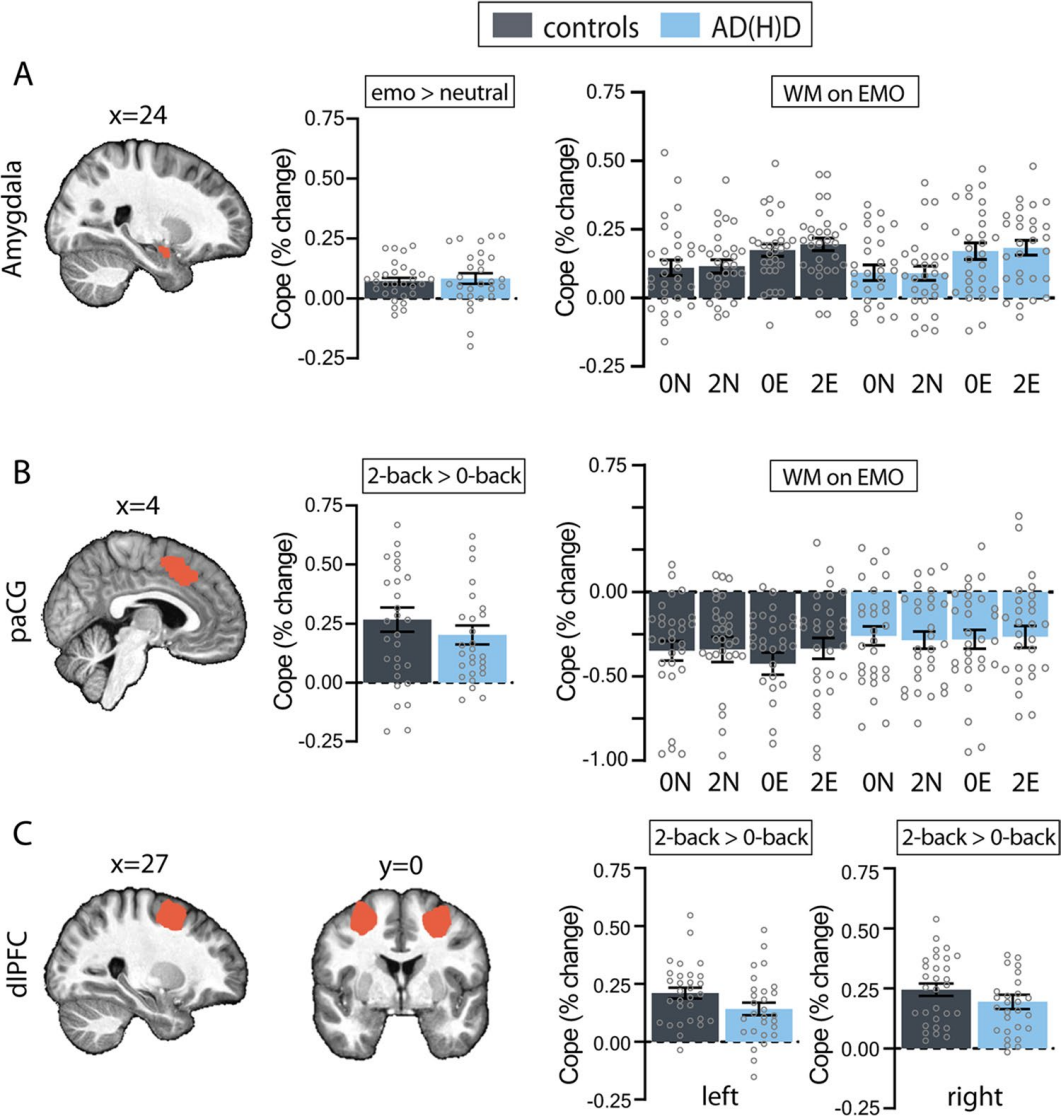
Region of Interest analysis: **A**) Amygdala activity during negative vs. neutral images (left) and emotional images preceded by working memory blocks (right) divided into the four conditions (0N, 2N, 0E, 2E). **B**) PaCG activity during 2-back vs. 0-back blocks (left) and emotional images preceded by working memory blocks (right) divided into the four conditions (0N, 2N, 0E, 2E). **C**) Left (left) and right (right) dlPFC activity during 2-back vs. 0-back blocks. paCG = paracingulate gyrus; dlPFC = dorsolateral prefrontal cortex; Error bars represent the standard error

In line with the whole brain results, we found no significant interaction between WM-load and group on PaCG activation and left dlPFC (PaCG: χ^2^(1) = 2.01, p = 0.15, ∆BIC =  − 2.73; all other ∆BIC < -4.74; left dlPFC: χ^2^(1) = 2.62, p = 0.11, ∆BIC =  − 2.13), but a main effect of WM-load was found (PaCG: χ^2^(1) = 41.12, p < 0.001, ∆BIC = 36.38; left dlPFC: χ^2^(1) = 56.35, p < 0.001, ∆BIC = 51.605)(Fig. 4A). Thus, high load WM-blocks (two-back) elicited more activity in the PaCG and left dlPFC than zero-back blocks, regardless of group. In the right dlPFC, we found a trend-significant interaction between group and WM-load (χ^2^(1) = 4.10, p = 0.04, ∆BIC =  − 0.65; all other ∆BIC <  − 3.73), indicating that there was less WM-related right dlPFC activity in participants with ADHD compared to controls, which suggests that WM processes are only limitedly impaired in the investigated ADHD population.

We assessed the effects of the emotional image type and group on amygdala activity during emotional processing (Fig. 4B). Model comparisons revealed evidence for a main effect of emotional images (χ^2^(1) = 37.32, p < 0.001, ∆BIC = 45.65), showing higher amygdala activity for negative than neutral images, but this was not moderated by group. We furthermore found no significant three-way interactions between emotional image type, preceding WM-block, and group (χ^2^(1) = 1.40, p = 0.84, ∆BIC =  − 20.37; all other ∆BIC <  − 5.19), which suggests that taxing WM did not influence ER-related amygdala activation.

Additionally, we assessed the effects of emotional images, the preceding WM-load and group on paCG activity during emotional processing, but found no three- or two-way interactions (χ^2^(1) = 3.66, p = 0.45, ∆BIC =  − 18.10; all other ∆BIC <  − 4); neither did we find a main effect of emotional images (χ^2^(1) = 0.97, p = 0.32, ∆BIC =  − 4.47), which suggests, in contrast to our hypothesis, paCG activity did not react to emotional salient stimuli, in either the ADHD or control group.

## Discussion

The primary aim of this study was to investigate whether taxing WM could ameliorate ER in adults with ADHD, and how WM and emotional reactivity (ER) would interact on a neural level. We demonstrated a significant main effect of negative emotional images on amygdala activation and a significant main effect of WM-load on activation of the paCG and dlPFC across both groups but did not find strong evidence for group differences. These findings were in line with the WM-task performance, which also did not reveal any group differences. Contrary to our hypothesis, neither amygdala nor paCG activity was reduced in response to negative vs. neutral images after inducing a high WM-load in participants with ADHD or controls.

### Emotional dysregulation in ADHD

ED in ADHD consist of a complex combination of dimensions, including emotion recognition, ER, and emotional lability. In the current study, however, we did not find amygdala hyperactivity in response to negative emotional stimuli in adults with ADHD. This contrasts earlier findings of amygdala hyperactivity in response to negative emotional faces (Brotman et al., 2010; Posner, et al., 2011a, 2011b; Quinlan et al., 2017) and related to delay aversion (Lemiere et al., 2012; Van Dessel et al., 2018, 2019) in children and adolescents with ADHD and in response to loss of anticipation (Tanaka et al., 2018; Wilbertz et al., 2017) and delayed rewards (Plichta et al., 2009) in adults with ADHD. Interestingly, the only two other studies that investigated ER in adult ADHD using stimuli similar to ours (i.e., IAPS images), also failed to find differences between participants with ADHD and controls (Hägele et al., 2016; Tajima-Pozo et al., 2018). In our study, valence ratings of the IAPS images by participants with ADHD were comparable to that of controls, implying that the lack of amygdala hyperactivity was not due to the lack of experiencing the images as negative. As such, amygdala hyperactivity in participants with ADHD might be related to deficits in the processing of specific negative stimuli (e.g., loss anticipation), instead of general deficits in emotion regulation. This is in line with an earlier notion that ER, as an aspect of ED in participants with ADHD, is influenced by many factors including age and is much more complex than originally thought (Graziano & Garcia, 2015).

### Working memory dysfunction in ADHD

In line with previous literature, we found that paCG and dlPFC were activated during the WM-task (high vs. low WM-load) (Müller & Knight, 2006; Roth & Courtney, 2007), but we did not find group differences in WM-related left dlPFC and paCG activation or behavior. In the right dlPFC, however, we found a trend-significant interaction between group and WM-load, suggesting blunted WM-related right dlPFC activation in participants with ADHD, which is consistent with other studies in adults (Cortese et al., 2012; Burgess et al., 2010; Ko et al., 2013), and adolescents with ADHD (Fassbender et al., 2011; Massat et al., 2012; Mattfeld et al., 2016; Prehn-Kristensen et al., 2011). In general, studies in adults with ADHD found less differences in neural recruitment between participants and controls than those in children with ADHD (Cortese et al., 2012). This suggests compensatory effects with age that has been proposed to involve parietal, occipital, and subcortical structures (i.e., cerebellum and basal ganglia) that may overcome deficits presented earlier in life (Cortese et al., 2012; Frazier et al., 2007). These compensatory mechanisms could explain why we did not find strong evidence for WM-dysfunction and associated frontal hypoactivity in adults with ADHD (Konrad & Eickhoff, 2010; Schweitzer et al., 2000, 2004). Nevertheless, previous studies have been inconsistent in their operationalization of WM tasks (e.g., n-back, recognition), assessing multiple aspects of working memory. As a possible contributor, differences in task complexity and difficulty of different tasks may have affected the extent of activation, and may even have activated different brain regions (Müller & Knight, 2006; Roth & Courtney, 2007). As this makes it challenging to compare results, we suggest that future studies take the complexity of WM tasks into account by including WM tasks that place greater demand on executive components of WM.

### Effect of working memory activation on emotional reactivity

Behaviorally, we found that participants recognized negative emotional images better but had worse recognition of images that were preceded by high-load WM-blocks, indicating an influence of WM-load on emotional memory processes. In the neuroimaging data, however, the amygdala and paCG activation were not reduced in response to negative emotional images when they were preceded by high WM-load tasks, suggesting no influence of WM-load on the neural correlates of ER. Previous studies that aimed to identify an interaction between WM and ER in controls and other patient populations used emotional interference tasks in which WM-tasks were performed *during* the presentation of neutral and negative emotional stimuli. Using such paradigms, high WM-load reduced amygdala activation to negative emotional stimuli in participants with an increased risk of ED (Richter et al., 2013). Also, negative emotional stimuli enhanced WM-related dlPFC activity in participants with major depressive disorder (Kerestes et al., 2012), adolescents with ADHD (Passarotti et al., 2010), and control adolescents, but not in control adults (Mueller et al., 2017). These effects were supported by impaired WM performance in presence of negative emotional stimuli in adolescents (López-Martín et al., 2013; Marx et al., 2011; Villemonteix et al., 2017) and adults with ADHD (Marx et al., 2014). Together, these studies suggest interaction effects between WM-load and ER-related amygdala (hyper-) activity. We were, however, unable to replicate these findings using a paradigm in which high WM-load was induced *prior to* emotional stimulation, instead of *during* the presentation of emotional stimuli. While our negative findings may, at least in part, be due to the limitations of our paradigm, an additional explanation is that improving ER by inducing high WM-loads will only be effective when severe impairments of ER are present. This may not always have been the case in our ADHD sample, as no group differences were found in amygdala reactivity to emotional images and only weak evidence was found for right dlPFC hypoactivity. Divergent emotional processing in ADHD has been linked to a variety of features in individuals with ADHD (Beheshti et al., 2020), such as externalizing symptoms, including conduct problems (Gillberg et al., 2004) and internalizing symptoms, such as depression and anxiety symptoms (Jarrett & Ollendick, 2008; Sciberras et al., 2014). The participants with ADHD we included here showed relatively low scores on symptoms of anxiety and depression (although higher than the controls), which provides an indication that ER capacities may not have been strongly affected in the studied population. Indeed, other studies that found amygdala hyperactivity in individuals with ADHD also reported higher symptoms of depression and anxiety in their study populations (Maier et al., 2014; Wilbertz et al., 2013). Interestingly, recent studies demonstrated that different subtypes of ED exist and that not all individuals with ADHD experience ED in all its complexities (Hirsch et al., 2019). This thus yields the possibility that our current sample of participants with ADHD may have only relatively mild impairments in ER.

As the dimensions of ED in adult ADHD are complex and multifaceted (Beheshti et al., 2020), it is essential to develop paradigms that can disentangle the effects of executive and emotional processes in adult ADHD. Our novel fMRI-task design allows us to investigate the interactions of WM and emotional processes, like ER, which may help to understand the underlying neural mechanisms and develop novel training for ADHD.

## Conclusion

We assessed possible neural correlates of the effects of targeting WM taxing on ER in adults with ADHD. We found no group differences in response to the emotional blocks on amygdala activation nor of WM-load on paCG and dlPFC activation. Although studies suggested an interaction between WM-load and emotional stimuli on the amygdala (hyper-) activity, we could not replicate these findings using our paradigm. These results might be due to compensatory effects in the adult participants with ADHD. Furthermore, targeting WM might still be effective in individuals with severe impairments in emotion regulation. These findings contribute to the understanding of the neural mechanisms of ER in controls and participants with ADHD.

## Supplementary Information

Below is the link to the electronic supplementary material.

Supplementary file1 (DOCX 851 kb)

## Data Availability

The data that support the findings of this study are available from the corresponding author upon reasonable request.

## Code availability

The code that supports the findings of this study are available from the corresponding author upon reasonable request.
