# Supplementary material for: Targeting working memory to modify emotional reactivity in adult attention deficit hyperactivity disorder: a functional magnetic resonance imaging study
Source: Brain Imaging Behav. 2021 Sep 15;16(2):680–91. doi: 10.1007/s11682-021-00532-6 (PMC9010388; doi:10.1007/s11682-021-00532-6)
Supplement: Supplementary file 1 — Supplementary file1 (DOCX 851 kb) [file 11682_2021_532_MOESM1_ESM.docx]

## Supplementary Materials

### 1. Supplementary methods

***1.1 Pilot study***

Seventeen healthy male control participants between the ages of 20 and 29 years old were included in a pilot study before the main study, with the same in- and exclusion criteria as for the controls of the main study. Based on that study, it was concluded that a sample of 30 male participants with ADHD and 30 matched healthy male controls would be sufficient to demonstrate a relevant effect of the task.

***1.2 fMRI task***

The following images from the IAPS database were used for the fMRI task.

| **Negative images** | old | 3053,3102,3080,3131,3000,3170,3064,3120,3015,3001,3100,9040,3130,3060,3069,3110,9410,3261,3530,3350,3071,3225,9405,9940,9810,9220,9910,9340,9635.1,9295,9901,9921 |
| --- | --- | --- |
|  | new | 7380,9520,9902,9920,9830,9904,9903,9301,6230,6021,9900,9000,9911,9031,3010,3150 |
| **Neutral images** | old | 2396,4559,7365,3302,7043,2122,2377,2397,2038,2690,8466,2351,8001,2411,2488,2309,2770,2191,2032,2514,2749,8032,2273,7255,7161,7012,7034,7081,7092,7207,7233,7020 |
|  | new | 7059,7001,7632,7021,5740,7036,7044,7179,5533,7014,7017,7033,7493,7513,2092,8160 |

**Table 1**: Images used from the IAPS database.

The experimental fMRI paradigm comprised a blocked design wherein active blocks with either the zero-back or the two-back task (‘WM block’) were interleaved with passive blocks consisting of either emotionally neutral or emotionally negative pictures (‘EMO block’). This resulted in four conditions: a two-back block followed by negative pictures (2E), a two-back block followed by neutral pictures (2N), a zero-back block followed by negative pictures (0E), and a zero-back block followed by neutral pictures (0N). The order of the blocks was randomized per participant, under the conditions that 1) the experiment started with a WM block and 2) blocks were never immediately followed by a block of the same type.

A WM block consisted of 15 letters that were shown on the screen one by one. During the zero-back condition, participants were instructed to use their index finger to press a button on the left response box when they saw an ‘x’ and to press a button on the right response box when they saw any letter other than ‘x’. During the two-back condition, participants were instructed to press the left button when the letter on the screen was the same as two letters before and the right button when the letter on the screen was different from two letters before. A total of 64 pictures shown during the experiment (32 emotionally negative and 32 emotionally neutral) were retrieved from the International Affective Picture System (IAPS). Every condition (2E, 2N, 0E, 0N) was shown twice, resulting in 16 blocks. The WM blocks lasted 30 s each, the EMO blocks 24s each (8 stimuli with a duration of 2.5s and 0.5s inter-stimulus interval). Instructions before and in between the blocks were 5s long. In total, the task was 8:53 min long. The percentage of correct responses in the n-back task were extracted and compared between conditions and group.

***1.3 Recognition task***

All participants performed a recognition task after the fMRI experiment to determine whether both groups paid equal attention to the images while conducting the fMRI task. Images were shown on a computer screen one-by-one, and participants were requested to indicate on a scale from 0 to 10 whether they recognized the pictures from the fMRI-task. A rating of 0 indicated they were confident they did not recognize the picture, a rating of 10 indicated they were confident they recognized the picture, and a rating of 5 indicated they were uncertain whether they recognized the picture. The recognition task included the 64 pictures that were shown during the fMRI experiment (‘old’ images) plus 32 ‘new’ images (16 emotionally neutral and 16 emotionally negative) also retrieved from the IAPS and meeting the same criteria as the other images. The recognition data were analyzed to obtain d-prime (d’) as a measure of discriminability, which is unaffected by response bias (Macmillan, 1993). The rating scale was first divided into recognized as new (0-4), unsure (5), and recognized as old (6-10) per participant. Trials with a rating of unsure were discarded for the analysis. To calculate d’, the z score that corresponds to the false-alarm rate is subtracted from the z-score that corresponds to the hit-rates (Macmillan, 1993). The recognition task data were binned into the task conditions of the fMRI task, resulting in d’ values per participant for 0N, 2N, 0E, and 2E. To correct for possible ceiling effects, the log-linear approach was used (Hautus, 1995).

***1.4 Validation task***

After the recognition task, the participants were requested to assess their subjective arousal in response to the images to measure the validity of the stimuli in our sample. During this validation task, participants rated the valence of all the images shown to them during the recognition task using the Self-Assessment Manikin (SAM) rating from 1 to 9, with one being ‘negative’ and nine being ‘positive’ (SAM; (Lang, 1980)). Ratings of all neutral and negative images were averaged per participant, respectively.

***1.5 fMRI analysis***

*Preprocessing with fMRIprep:*

Preprocessing was performed using FMRIPREP v1.2.3. Each T1w scan was bias-corrected, skull-stripped, and subsequently normalized to MNI space using non-linear registration. Functional data preprocessing included motion correction using FLIRT and distortion correction using an implementation of the TOPUP technique using 3dQwarp. This was followed by co-registration to the corresponding T1w using boundary-based registration with 9 degrees of freedom. Motion correcting transformations, field distortion correcting warp, BOLD-to-T1w transformation, and T1w-to-template (MNI) warp were concatenated and applied in a single step using antsApplyTransforms (ANTs v2.1.0) with Lanczos interpolation. Independent component analysis (ICA) based on Automatic Removal Of Motion Artifacts (AROMA) was used to generate data that was non-aggressively denoised. Subsequently, data were spatially smoothed (6mm FWHM), and a high pass-filter (342s) was applied using FSL. Since amygdala fMRI signals may be particularly affected by susceptibility artifacts induced by the magnetic field inhomogeneities in the ventral part of the brain, we calculated temporal signal-to-noise-ratio (tSNR) maps for the whole brain (Supplementary Results 2.1).

The following design matrices are examples of the ones used in this study.


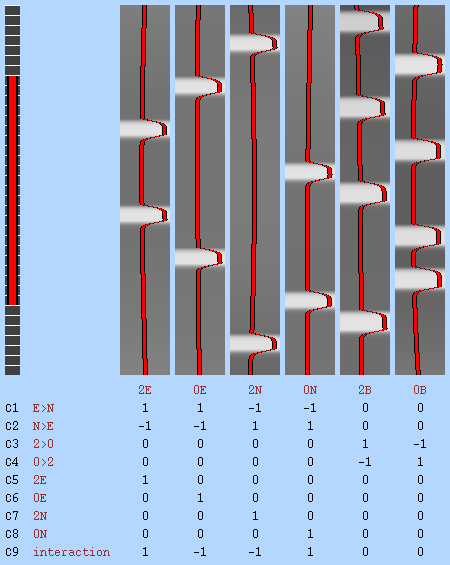

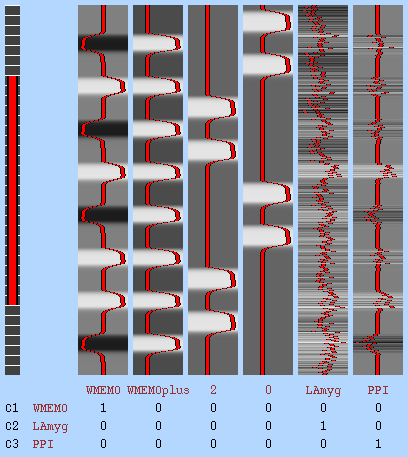


**Supplementary Figure 1**: **A)** Design Matrix of one representative participant for the task-based analysis. The model was designed to estimate the effect of working memory load on the neural correlates of emotional processing. **B)** Design Matrix of one representative participant for the PPI analysis. The model included the timeseries of the ROI as well as the task regressors and the PPI regressor.

*ROI analysis*

To assess the effect of WM-load on emotional processing, ROI analyses were conducted using predefined ROIs. The left and right amygdala were chosen as ROIs because they are key regions in emotional processing and were extracted from the Harvard-Oxford Subcortical Structural Atlas with a threshold of 90%. The dlPFC and the paCG were chosen as ROIs as they play a key role in executive functioning, especially during n-back tasks. Additionally, overlapping activation in studies using negative affect and cognitive control paradigms was found in the paCG. The dlPFC and paCG masks were based on the most robust activated clusters from a meta-analysis across 1091 WM fMRI studies (Neurosynth). For the amygdala, hemisphere differences were tested using a paired t-test and found not to be significant. Therefore, all parameters were averaged across hemispheres. For the dlPFC, hemisphere differences were tested using a paired t-test and found to be significant. The left and right dlPFC were, therefore, analyzed separately. Featquery (FSL) was used to extract the COPEs, which were converted to a percentage change.

***1.6 Statistical Analysis***

To assess all data for normality, histograms, and QQplots were inspected, and a Shapiro-Wilk test was performed. In the case of a non-normal distribution, a transformation was applied. Data points that were more than three standard deviations from the mean were removed as outliers. Demographics were analyzed with SPSS v.23 (IBM, Chicago, USA). Normally distributed data were tested using independent t-tests; otherwise, a Whitney-U test was used. To account for more than one source of random variability within-participant and across-participants, analyses of the task and fMRI data used linear mixed-effects models in Rv.3.5.3 (R Development Core Team, 2011) using the lme4 package (Bates, Mächler, Bolker, & Walker, 2015). The main and interaction effects of emotional load (EMO: emotional/neutral), working memory (WM: 2-back/0-back), and group (GRP: ADHD/control) were assessed as fixed effects. The model selection process was based on an adjusted top-down procedure, where the full model (three-way interaction) was tested based on the Akaike information criterion (AIC) using the step function, which resulted in the most complex fixed effects structure that would then further be tested.

A random effect per subject was always included in the model (SUBJECT|1), controlling for individual variation among participants. The simplest model was then increased in complexity, adding fixed effects until all fixed effects that resulted from step one were included and compared using the Bayesian information criterion (BIC) (Fabozzi, Focardi, Rachev, & Arshanapalli, 2014; Schwarz, 1978). Random slopes were then added and tested based on BICs and χ² tests. If these were found to add in explaining the variance, they were added as random effects, and all fixed effects were tested again based on the described semi top-down strategy. The model found best capturing the data was consequently reported and compared to other models using χ² tests and BICs. All statistical tests were conducted using a significance level of p < 0.05. ∆BIC is calculated by subtracting the BIC value of the tested model from the model it is being compared to. A negative ∆BIC, therefore, indicates that the tested model did not explain the variance better, whereas a positive ∆BIC indicates that the tested model did explain the variance better. A ∆BIC between 0 and 2 can be interpreted as weak evidence, 2-6 as positive evidence, 6-10 as strong evidence, and ∆BIC>10 as very strong evidence against the model with the higher BIC value (Raftery, 1995).

**1.7 Psychophysiological Interaction (PPI) analysis**

To investigate the effect of WM-load during emotional processing on the connectivity of the ROIs (left and right amygdala, dlPFC, and paCG) to the rest of the brain, a PPI analysis was conducted. ICA-AROMA denoised functional data were entered into the first-level analysis (FSL/FEAT v.6.00), which included the time-series per ROI as well as the task regressors and the PPI regressor (Supplementary Figure 1). Whole-brain analyses were performed as described above, using the first level data of the PPI analysis.

**Supplementary Figure 2**: Temporal signal-to-noise analysis. **A)** Whole brain tSNR maps for ADHD patients (up) and controls (down). **B)** Average tSNR per participant (circles) and group (bars) per ROI and for both ADHD patients (blue) and controls (grey). paCG = paracingulate Gyrus, dlPFC = dorsolateral prefrontal cortex.


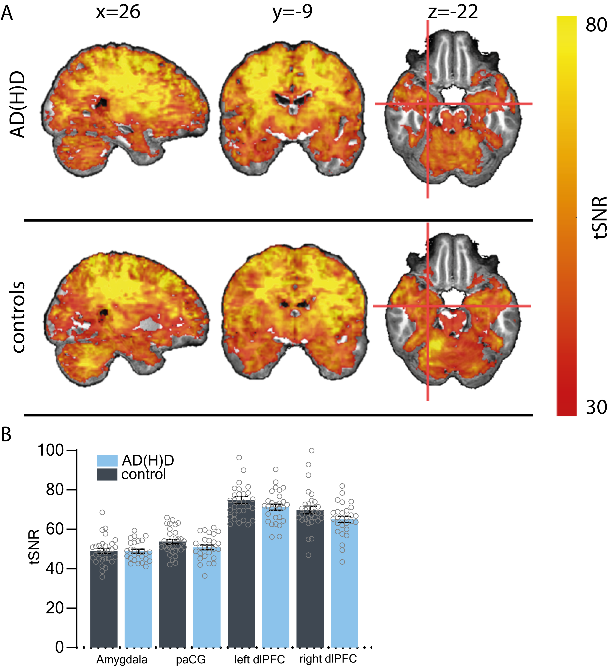


### 2. Supplementary results

***2.1 Temporal Signal-to-Noise (tSNR)***

Since amygdala fMRI signals may be particularly affected by susceptibility artifacts induced by the magnetic field inhomogeneities in the ventral part of the brain (Merboldt, Fransson, Bruhn, & Frahm, 2001), we calculated tSNR maps for the whole brain (Welvaert & Rosseel, 2013).

The average tSNR in the amygdala for participants with ADHD was 48.88(5.05), and for controls was 48.96(6.83). In the paCG, the average tSNR for participants with ADHD was 50.8(6.29), and for controls was 53.71(6.57). In the left dlPFC, the average tSNR for participants with ADHD was 71.21(8.12), and for the controls was 74.74(10.34), in the right dlPFC, it was 64.95(8.41) for participants with ADHD and 69.79(10.43) for controls. As previously reported, a tSNR of around 50 requires the acquisition of around 350 volumes to detect a 2% signal change (Murphy, Bodurka, & Bandettini, 2007). We acquired 370 volumes. We could, therefore, conclude that we had sufficient tSNR to measure activation in our regions of interest.

***2.2 Whole Brain Analysis – Cluster Table***

| Contrast | Region | Cluster Index | Voxels | MAX | MAX X (vox) | MAX Y (vox) | MAX Z (vox) | COG X (vox) | COG Y (vox) | COG Z (vox) |
| --- | --- | --- | --- | --- | --- | --- | --- | --- | --- | --- |
| Negative images > neutral images | | |  |  |  |  |  |  |  |  |
|  | Occipital Cortex, Amygdala, Left Thalamus | 5 | 39784 | 1 | 27 | 42 | 22 | 44.5 | 37.2 | 33.4 |
|  | Superior Frontal Gyrus | 4 | 1210 | 0.994 | 48 | 90 | 47 | 45.3 | 91.7 | 47.2 |
|  | Right Precentral Gyrus | 3 | 308 | 0.959 | 19 | 64 | 47 | 19.8 | 66.6 | 48.4 |
|  | Right Precentral Gyrus | 2 | 162 | 0.957 | 25 | 59 | 58 | 22.8 | 61.1 | 59.5 |
|  | Paracingulate Gyrus | 1 | 10 | 0.951 | 44 | 89 | 34 | 44.2 | 88.5 | 34.8 |
| 2-back > 0-back | |  |  |  |  |  |  |  |  |  |
|  | Frontal Pole, Inferior Frontal Gyrus, Middle Frontal Gyrus, Paracingulate Gyrus | 5 | 34443 | 1 | 27 | 91 | 28 | 44.2 | 59.2 | 54.8 |
|  | Cerebellum | 4 | 8119 | 1 | 29 | 27 | 7 | 44.9 | 30.2 | 18.9 |
|  | Left Inferior Temporal Gyrus | 3 | 287 | 0.986 | 71 | 34 | 31 | 70.1 | 34.9 | 32 |
|  | Right Inferior Temporal Gyrus | 2 | 141 | 0.982 | 18 | 38 | 31 | 17.4 | 37.9 |  |
|  | Brain Stem | 1 | 15 | 0.955 | 44 | 46 | 35 | 43.5 | 47.4 |  |

**Supplementary Table 2**: Clusters resulting from the whole brain analysis, assessing the effects of working memory load and emotional stimulus type.

***2.3 Habituation Analysis***

A habituation analysis of the emotional response within the amygdala was performed to exclude the possibility of a difference in adaptive reduction in response to the emotional stimuli between participants with ADHD and controls. Blocks of emotional stimuli were analyzed dependent on when they were presented (N1-N4/E1-E4). With the numbers indicating the position of the block in time. For the negative stimulus type there was no interaction effect of group and time found (χ²(3) = 4.85, p = 0.18, ∆BIC = -11.5). Also, no main effects of group or time were found (all other ∆BIC < -8.35). For the neutral stimulus type there was no interaction effect of group and time found (χ²(3) = 1.13, p = 0.77, ∆BIC = -15.01). Also, no main effects of group or time were found (all other ∆BIC < -5.24). We can, therefore, conclude that there was no habituation effect to negative or neutral emotional stimuli in the amygdala, for neither participants with ADHD nor controls.

**Supplementary Figure 3**: Habituation analysis. **A**) Amygdala activity in response to the first (N1), second (N2), third (N3) and fourth (N4) block of neutral images, for ADHD patients (blue) and controls (grey). **B)** Amygdala activity in response to the first (E1), second (E2), third (E3) and fourth (E4) block of negative images, for ADHD patients (blue) and controls (grey).


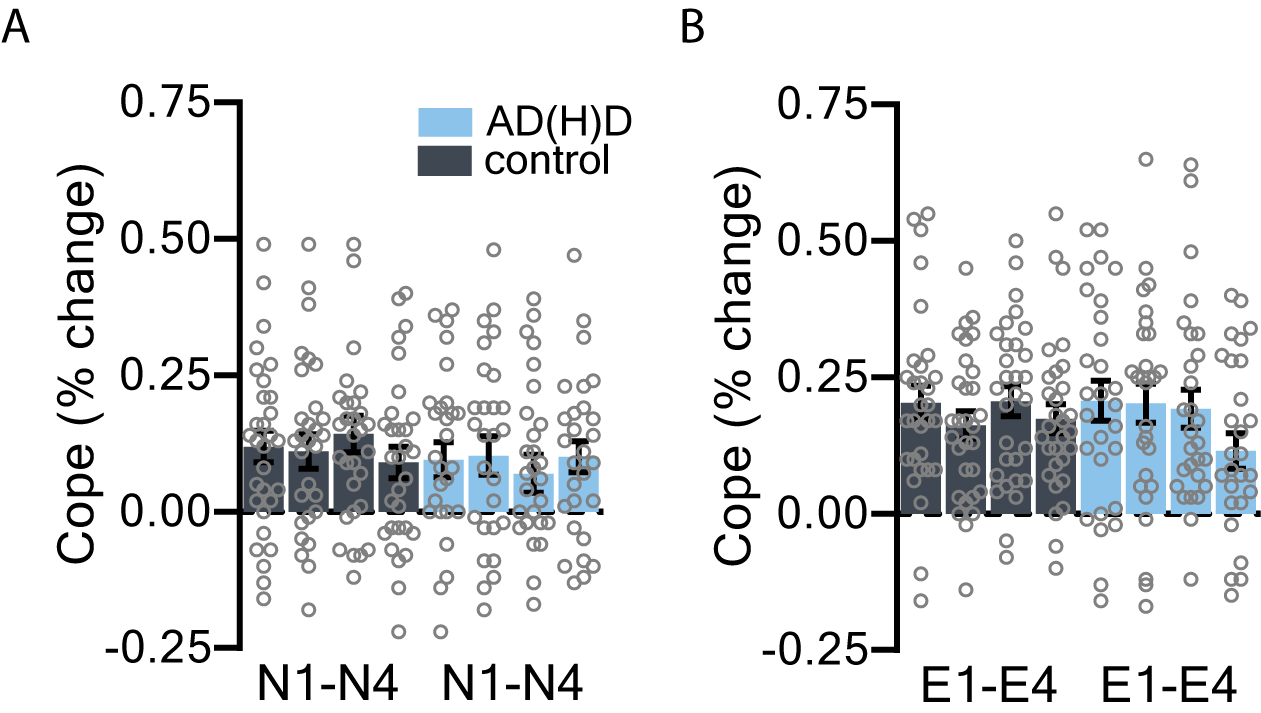


***2.4 Heartrate analysis***


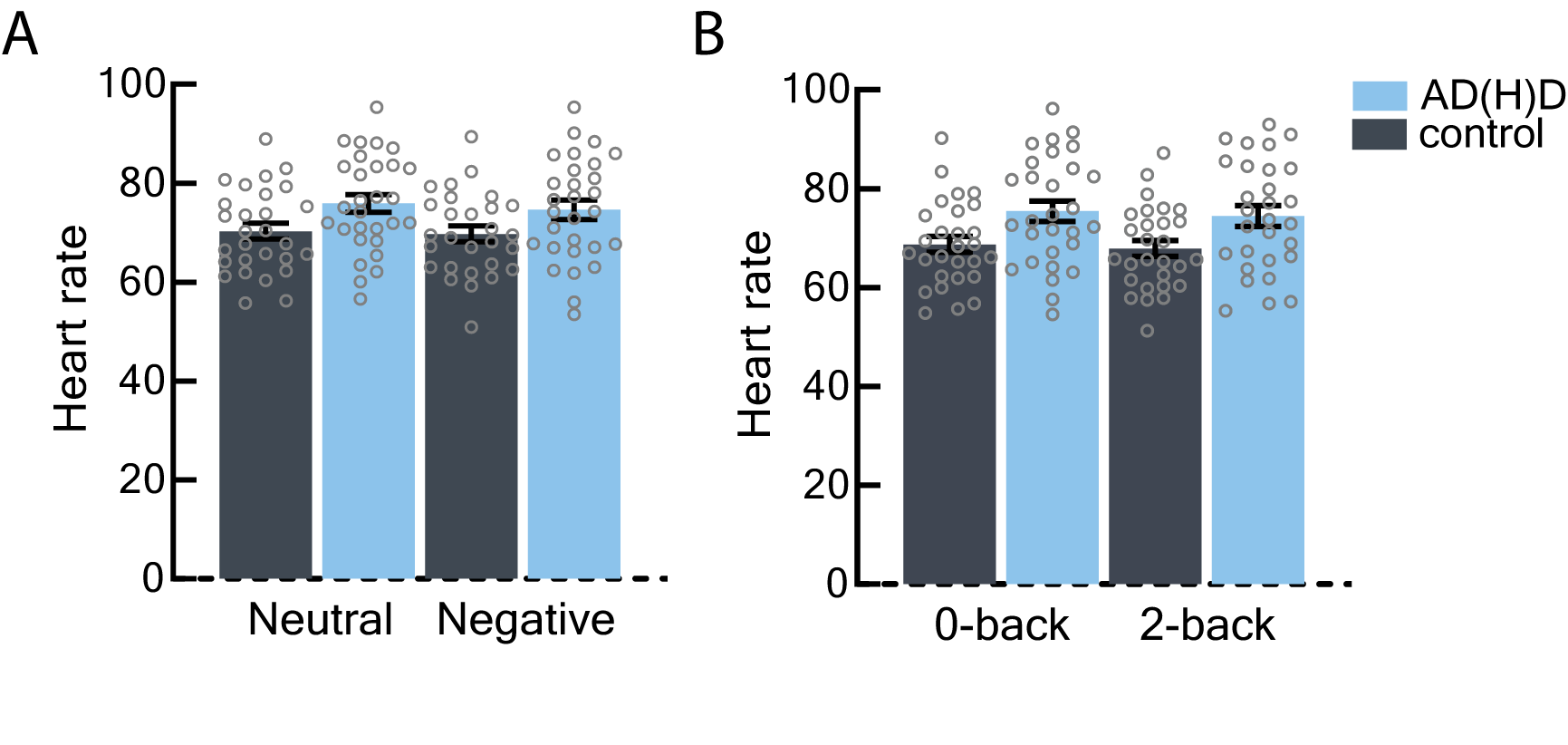
Heart rate was measured during scanning using a PPU to obtain plethysmographs. The average heart rate per WM load and type of emotional stimulus was calculated using MATLAB scripts. For the heart rate during the working memory task, no interaction effect of group and working memory load could be found (∆BIC < -2.76). A main effect of group was found (HR ~ GRP + (1|SUBJECT); χ²(1) = 5.70, p < 0.02, ∆BIC = 1.02). For heart rate during emotional stimuli, no interaction effect of group and type of emotional stimulus were found (∆BIC < -0.16). Also, no main effects of group or emotional stimuli were found, even though there was a trend towards a group effect. This indicates that participants with ADHD have a higher heart rate, independent of which task they were doing. We can conclude that the task had no different effect on participants with ADHD than on controls. participants with ADHD demonstrated a higher heart rate in general, which could be an effect of ADHD medication.

**Supplementary Figure 4**: Heartrate analysis. **A)** Heartrate of participants with ADHD(blue) and controls (grey) during the presentation of neutral and negative emotional stimuli. **B)** Heartrate of participants with ADHD(blue) and controls (grey) during the low-load (0-back) and high-load (2-back) working memory task.

***2.5 PPI***

*Whole Brain*

For the whole brain PPI analysis, we assessed the effects of WM-load, emotional stimulus type, and group. Permutation tests did not show any connectivity of the left and right amygdala, nor paCG or left or right dlPFC with the rest of the brain. When contrasting emotionally negative and neutral images and high versus low WM-load, we found no differences in connectivity of the paCG or left or right dlPFC with the rest of the brain. Furthermore, we found no interaction effect of WM-load and emotional stimulus type on the connectivity of the left and right amygdala nor paCG or left or right dlPFC with the rest of the brain. Neither did we find any group differences.

In line with the presented task effects, no group differences were found, which could be explained by a low impairment of ER in our current ADHD patients. Positive structural and functional amygdala-frontal coupling as a mechanism that provides top-down control of emotions has been shown, e.g., in controls (Banks, Eddy, Angstadt, Nathan, & Luan Phan, 2007; Ochsner et al., 2009), during emotion regulation tasks (Stein et al., 2007) and more specifically as a top-down inhibitory effect of the PFC on the amygdala (Banks et al., 2007). Moreover, studies using PPI-based analyses showed increased co-activation between the amygdala and the medial PFC (Erk, Abler, & Walter, 2006; Schmitz & Johnson, 2006; Williams et al., 2006) and between the amygdala and the dlPFC in depression (Siegle, Thompson, Carter, Steinhauer, & Thase, 2007) during cognitive-emotional tasks. In contrast, Hägele et al., who also used IAPS stimuli, did not find any PPI effects using a seed in the left and right amygdala, which they interpreted as due to high inter-individual differences in patients (Hägele et al., 2016). These differences, including, e.g., differences in perception of stimuli or different symptom severities, make it difficult to reach the necessary power to calculate a PPI in patient populations, and indicate that effect sizes in these kinds of analyses are very small (O’Reilly, Woolrich, Behrens, Smith, & Johansen-Berg, 2012).

### Methodological considerations:

To make sure our results were not due to methodological limitation, we assessed temporal signal-to-noise-ratio (tSNR) maps per participant, investigated habituation effects in the amygdala, and the influence by heartrate. Amygdala fMRI signals can be especially affected by susceptibility-artifacts induced by inhomogeneities in the subcortex (Merboldt et al., 2001). Therefore, we assessed temporal signal-to-noise-ratio (tSNR) maps per participant (Welvaert and Rosseel, 2013; Supplementary Results 2.1), which were sufficiently high in all ROIs for all participants. Additionally, amygdala activation to emotional stimuli has been suggested to show a different adaptive reduction between ADHD patients and controls (Breiter et al., 1996; Garrett et al., 2012). However, we did not find any evidence for such habituation effects (Supplementary materials and results 2.3). As BOLD contrast is based on hemodynamic changes, heart rate (HR) might cause fluctuations in the signal (Shmueli et al., 2007). Task effects were not found to be influenced by HR (supplementary materials and results 2.4). Therefore, it is unlikely that our results were affected by these methodological challenges.

### 3. References:

Banks, S. J., Eddy, K. T., Angstadt, M., Nathan, P. J., & Luan Phan, K. (2007). Amygdala-frontal connectivity during emotion regulation. *Social Cognitive and Affective Neuroscience*, *2*(4), 303–312. https://doi.org/10.1093/scan/nsm029

Bates, D., Mächler, M., Bolker, B., & Walker, S. (2015). Fitting Linear Mixed-Effects Models Using lme4. *Journal of Statistical Software*, *67*(1), 1–48. https://doi.org/10.18637/JSS.V067.I01

Breiter, H. C., Etcoff, N. L., Whalen, P. J., Kennedy, W. A., Rauch, S. L., Buckner, R. L., … Rosen, B. R. (1996). Response and habituation of the human amygdala during visual processing of facial expression. *Neuron*, *17*(5), 875–887. https://doi.org/10.1016/S0896-6273(00)80219-6

Erk, S., Abler, B., & Walter, H. (2006). Cognitive modulation of emotion anticipation. *European Journal of Neuroscience*, *24*(4), 1227–1236. https://doi.org/10.1111/j.1460-9568.2006.04976.x

Fabozzi, F. J., Focardi, S. M., Rachev, S. T., & Arshanapalli, B. G. (2014). Appendix E: Model Selection Criterion: AIC and BIC. In *The Basics of Financial Econometrics*. https://doi.org/10.1002/9781118856406.app5

Garrett, A. S., Reiss, A. L., Howe, M. E., Kelley, R. G., Singh, M. K., Adleman, N. E., … Chang, K. D. (2012). Abnormal amygdala and prefrontal cortex activation to facial expressions in pediatric bipolar disorder. *Journal of the American Academy of Child and Adolescent Psychiatry*, *51*(8), 821–831. https://doi.org/10.1016/j.jaac.2012.06.005

Hägele, C., Friedel, E., Schlagenhauf, F., Sterzer, P., Beck, A., Bermpohl, F., … Heinz, A. (2016). Affective responses across psychiatric disorders-A dimensional approach. *Neuroscience Letters*, *623*, 71–78. https://doi.org/10.1016/j.neulet.2016.04.037

Hautus, M. J. (1995). Corrections for extreme proportions and their biasing effects on estimated values ofd′. *Behavior Research Methods, Instruments, & Computers*, *27*(1), 46–51. https://doi.org/10.3758/BF03203619

Lang, P. (1980). Behavioral treatment and bio-behavioral assessment: Computer applications. *Technology in Mental Health Care Delivery Systems*, 119–137.

Macmillan, N. A. (1993). Signal detection theory as data analysis method and psychological decision model. In *A handbook for data analysis in the behavioral sciences: Methodological issues.* (pp. 21–57). Hillsdale, NJ, US: Lawrence Erlbaum Associates, Inc. https://doi.org/tbd

Merboldt, K.-D., Fransson, P., Bruhn, H., & Frahm, J. (2001). Functional MRI of the Human Amygdala? *NeuroImage*, *14*(2), 253–257. https://doi.org/10.1006/NIMG.2001.0802

Murphy, K., Bodurka, J., & Bandettini, P. A. (2007). How long to scan? The relationship between fMRI temporal signal to noise ratio and necessary scan duration. *NeuroImage*, *34*(2), 565–574. https://doi.org/10.1016/j.neuroimage.2006.09.032

O’Reilly, J. X., Woolrich, M. W., Behrens, T. E. J., Smith, S. M., & Johansen-Berg, H. (2012). Tools of the trade: Psychophysiological interactions and functional connectivity. *Social Cognitive and Affective Neuroscience*, *7*(5), 604–609. https://doi.org/10.1093/scan/nss055

Ochsner, K. N., Ray, R. R., Hughes, B., McRae, K., Cooper, J. C., Weber, J., … Gross, J. J. (2009). Bottom-up and top-down processes in emotion generation: common and distinct neural mechanisms. *Psychological Science*, *20*(11), 1322–1331. https://doi.org/10.1111/j.1467-9280.2009.02459.x

R Development Core Team, R. F. F. S. C. (2011). R: A language and environment for statistical computing.

Raftery, A. E. (1995). Bayesian Model Selection in Social Research. *Sociological Methodology*, *25*, 111–163.

Schmitz, T. W., & Johnson, S. C. (2006). Self-appraisal decisions evoke dissociated dorsal-ventral aMPFC networks. *NeuroImage*, *30*(3), 1050–1058. https://doi.org/10.1016/j.neuroimage.2005.10.030

Schwarz, G. (1978). Estimating the Dimension of a Model. *The Annals of Statistics*. https://doi.org/10.1214/aos/1176344136

Shmueli, K., van Gelderen, P., de Zwart, J. A., Horovitz, S. G., Fukunaga, M., Jansma, J. M., & Duyn, J. H. (2007). Low-frequency fluctuations in the cardiac rate as a source of variance in the resting-state fMRI BOLD signal. *NeuroImage*, *38*(2), 306–320. https://doi.org/10.1016/j.neuroimage.2007.07.037

Siegle, G. J., Thompson, W., Carter, C. S., Steinhauer, S. R., & Thase, M. E. (2007). Increased Amygdala and Decreased Dorsolateral Prefrontal BOLD Responses in Unipolar Depression: Related and Independent Features. *Biological Psychiatry*, *61*(2), 198–209. https://doi.org/10.1016/j.biopsych.2006.05.048

Stein, J. L., Wiedholz, L. M., Bassett, D. S., Weinberger, D. R., Zink, C. F., Mattay, V. S., & Meyer-Lindenberg, A. (2007). A validated network of effective amygdala connectivity. *NeuroImage*, *36*(3), 736–745. https://doi.org/10.1016/j.neuroimage.2007.03.022

Welvaert, M., & Rosseel, Y. (2013). On the Definition of Signal-To-Noise Ratio and Contrast-To-Noise Ratio for fMRI Data. *PLoS ONE*, *8*(11), e77089. https://doi.org/10.1371/journal.pone.0077089

Williams, L. M., Das, P., Liddell, B. J., Kemp, A. H., Rennie, C. J., & Gordon, E. (2006). Mode of functional connectivity in amygdala pathways dissociates level of awareness for signals of fear. *Journal of Neuroscience*, *26*(36), 9264–9271. https://doi.org/10.1523/JNEUROSCI.1016-06.2006
